# Corneal biomechanics are not exclusively compromised in high myopia

**DOI:** 10.1111/opo.13313

**Published:** 2024-04-02

**Authors:** Fabian Yii, Niall Strang, Miguel O. Bernabeu, Baljean Dhillon, Tom MacGillivray

**Affiliations:** 1 Centre for Clinical Brain Sciences, The University of Edinburgh, Edinburgh, UK; 2 Curle Ophthalmology Laboratory, Institute for Regeneration and Repair, The University of Edinburgh, Edinburgh, UK; 3 Department of Vision Sciences, Glasgow Caledonian University, Glasgow, UK; 4 Centre for Medical Informatics, Usher Institute, The University of Edinburgh, Edinburgh, UK; 5 The Bayes Centre, The University of Edinburgh, Edinburgh, UK; 6 Princess Alexandra Eye Pavilion, NHS Lothian, Edinburgh, UK

**Keywords:** corneal biomechanics, corneal hysteresis, corneal resistance factor, myopia

## Abstract

**Introduction:**

Research assuming linearity has concluded that corneal biomechanics are compromised in high myopia. We investigated whether this assumption was appropriate and re-examined these associations across different levels of myopia.

**Methods:**

Myopic (spherical equivalent refraction, SER ≤ −0.50 D) eyes of 10,488 adults aged 40–69 years without any history of systemic and ocular conditions were identified in the UK Biobank. Ordinary least squares (OLS) regression was employed to test the linear association between corneal hysteresis (CH) or corneal resistance factor (CRF), separately, and SER while controlling for age, sex, corneal radius and intraocular pressure. Quantile regression (QR) was used to test the same set of associations across 49 equally spaced conditional quantiles of SER.

**Results:**

In OLS regression, each standard deviation (SD) decrease in CH and CRF was associated with 0.08 D (95% CI: 0.04–0.12; *p* < 0.001) and 0.10 D (95% CI: 0.04–0.15; *p* < 0.001) higher myopia, respectively. However, residual analysis indicated that the linearity assumption was violated. QR revealed no evidence of a significant association between CH/CRF and SER in low myopia, but a significant (*p* < 0.05) positive association became evident from −2.78 D (0.06 and 0.08 D higher myopia per SD decrease in CH and CRF). The magnitude of association increased exponentially with increasing myopia: in the −5.03 D quantile, every SD decrease in CH and CRF was associated with 0.17 D (95% CI: 0.08–0.25; *p* < 0.001) and 0.21 D (95% CI: 0.10–0.31; *p* < 0.001) higher myopia. In the −8.63 D quantile, this further increased to 0.54 D (95% CI: 0.33–0.76; *p* < 0.001) and 0.67 D (95% CI: 0.41–0.93; *p* < 0.001) higher myopia per SD decrease in CH and CRF.

**Conclusions:**

Corneal biomechanics appeared compromised from around −3.00 D. These changes were observed to be exponential with increasing myopia.

**Supplementary Information:**

The online version of this article (doi:10.1111/opo.13313) contains supplementary material, which is available to authorized users.

## Key points

- Corneal biomechanics are compromised from around −3.00 D, with an exponential (non-linear) reduction in corneal hysteresis and corneal resistance factor as myopia increases thereon.
- These findings show that structural complications/sequelae of myopia are not specific to high myopia, and underscore the importance of not misconstruing the dioptric threshold of −6.00, or −5.00 D for that matter, as being intrinsically dichotomous (safe vs. unsafe threshold).
- The strong non-linear association between corneal biomechanics and myopia may be one important reason why some previous studies failed to find a significant association using conventional linear models, as the magnitude of association would be underestimated by such models (increasingly so with greater myopia).

## INTRODUCTION

While the exact aetiology of myopia remains elusive, experimental models of emmetropisation and refractive error development have significantly advanced our understanding of the mechanisms underlying myopic eye growth.^1^ One important contribution is the finding that early visual experience (optical defocus or form deprivation) influences the axial elongation rate in a highly predictable manner, brought about by a biochemical signalling cascade that originates locally in the retina, traverses the choroid and eventually remodels the scleral extracellular matrix.^1^ In myopia, this renders the scleral tissue more distensible (extensible), ultimately increasing the eye's susceptibility to excessive growth.^2–4^ In addition to being a key determinant of myopia development and progression, compromised scleral biomechanics are implicated in various sight-threatening pathological sequalae of myopia, such as posterior staphyloma.^5^

Although some studies have attempted to assess anterior scleral resistance using rebound tonometry,^6,7^ there are currently no validated, clinically available methods to measure the biomechanics of equatorial and posterior sclera in vivo. That said, indirect evidence from recent studies suggests that corneal biomechanics, which can be readily measured in vivo, may be used as a surrogate measure for the overall scleral biomechanics,^8–11^ a prospect not too far-fetched on account of the close histological link between the cornea and the sclera.^12^ Furthermore, our recent study^13^ showed that, in children with high axial myopia (and thus pronounced ocular compliance), higher Goldmann intraocular pressure (IOP) was associated with slower axial growth, further pointing towards a connection between anterior corneal biomechanics (which influence Goldmann applanation tonometry) and the overall compliance of the eye.

Driven in part by this, several studies^14–29^ have looked at the relationship between myopia and corneal biomechanical properties measured using an Ocular Response Analyser, ORA (reichert.com)—a non-contact tonometer that provides corneal hysteresis (CH) and corneal resistance factor (CRF) readings. ORA applies a rapid air pulse and records the response of the central cornea as it moves inwards past its first point of applanation, becoming slightly concave, before rebounding and moving past its second point of applanation.^30^ CH is the difference between the pressures measured at the first (P1) and second (P2) applanation, which is thought to reflect the ability of the cornea to dissipate energy, as the difference represents the amount of energy loss or absorption by its tissue during deformation.^11^ CRF is a parameter that reflects the overall corneal resistance to deformation (P1 + *k* × P2, where *k* is a constant derived empirically to make CRF correlate more strongly with central corneal thickness than CH).^31^ A lower CH and CRF would suggest that the cornea has reduced ability to dissipate energy and resist deformation (less rigid), respectively. The mean value of P1 and P2 represents IOP, which has been shown to correlate well with readings obtained using Goldmann applanation tonometry.^30,32^

Previous studies generally concluded that corneal biomechanics were compromised in high myopia (e.g., spherical equivalent refraction, SER < −6 D) compared with low myopia and emmetropia, as indicated by reduced CH or CRF,^14,15,18–24,27,29^ although a statistically significant result for the latter is less consistently reported.^15,21,24^ These studies, however, relied on linear regression or its variants such as analysis of variance and *t*-tests, all of which fall under general linear models and are limited by the implicit assumption that the relationship between CH/CRF and myopia was linear across a wide range of myopia severity. This constraint precluded elucidation of the nature of corneal biomechanical changes in myopia, such as whether the cornea in eyes with lower levels of myopia is also compromised and, if so, at what level and to what extent. Moreover, whether the direction and magnitude of these biomechanical changes remain constant across the full range of myopia remains an open question.

Assuming that corneal biomechanics are indeed reflective of the overall ocular/scleral biomechanics, one may hypothesise that corneal biomechanics are not exclusively compromised in eyes with high myopia, since pathologic myopia (including posterior staphyloma) can also be found in eyes with much lower levels of myopia.^33,34^ Besides, corneal biomechanical changes are likely to occur in a highly non-linear fashion with increasing myopia, considering that the odds of pathologic myopia and retinal detachment have been found to increase quasi-exponentially as myopia increases.^35^ The present study aimed to test these hypotheses by examining the relationship between CH/CRF and SER across different levels of myopia using a flexible statistical modelling technique in a large population-based cohort of healthy adults.

## METHODS

### Participant selection criteria

The UK Biobank is a population-based cohort of half a million adults residing in the United Kingdom, all of whom participated in a wide range of baseline assessments between 2006 and 2010. The database is linked to the National Health Service electronic health records and national death registries. As the UK Biobank has a prior Research Tissue Bank approval from the North West Multi-Centre Research Ethics Committee (06/MRE08/65), a separate ethical clearance was not required for the present study.

**FIGURE 1:**
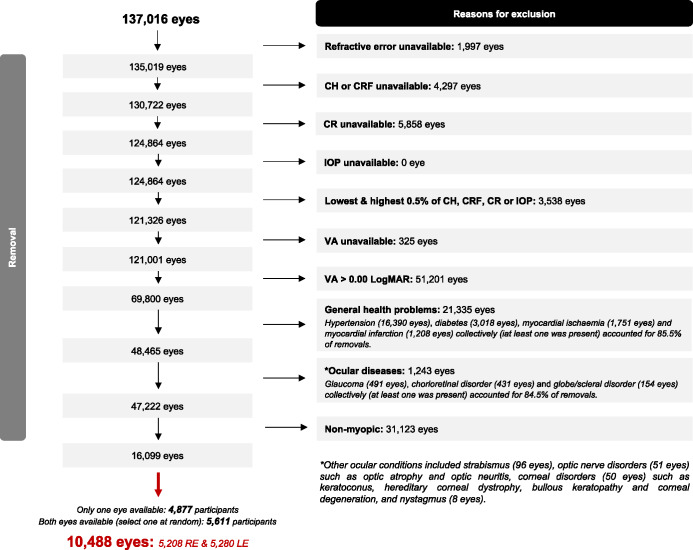
Flow diagram detailing each step of the participant selection process. CH, corneal hysteresis; CR, corneal radius of curvature; CRF, corneal resistance factor; IOP, intraocular pressure; LE, left eye; RE, right eye.

A total of 68,508 phakic participants additionally underwent a standardised ophthalmic assessment at baseline.^36^ The flow diagram in Figure 1 details how the participants included in the present study were selected. Briefly, eyes with missing refractive error, CH, CRF, corneal radius of curvature (CR) or IOP data were excluded because these measurements were not taken in participants who had undergone any eye surgery within the last 4 weeks or presented with possible signs of ocular infection. We then removed extreme values (top and bottom 0.5%) of CH, CRF, CR and IOP because these were most likely caused by measurement error. Eyes with missing distance visual acuity (VA) data or poor VA (>0.00 LogMAR) were further removed. Using linked healthcare data derived from a combination of primary care records, hospital inpatient admissions, death registries and medical questionnaires administered as part of the baseline assessment,^37^ we identified participants with a history of chronic or severe health condition(s)—which included cardiovascular diseases (hypertension, myocardial infarction/ischaemia, heart failure, cardiomyopathy, cardiac arrest, heart valve disease, atherosclerosis), cerebral infarction, diabetes, thalassaemia/anaemia, sickle cell disease, rheumatic diseases, sarcoidosis, Down syndrome, Edwards' syndrome, Patau's syndrome, Turner syndrome, Marfan syndrome, Ehlers–Danlos syndrome and spina bifida. Both eyes of these participants were removed, with hypertension, diabetes and/or myocardial infarction/ischaemia contributing to 85.5% of all removals. Eyes with ocular diseases including keratoconus and hereditary corneal dystrophy were further removed (similarly identified via linked healthcare data), 84.5% of which were removed due to glaucoma, chorioretinal disorders and/or globe/scleral disorders such as degenerative myopia and staphyloma. Of the remaining 47,222 eyes, 16,099 eyes with myopia (SER ≤ −0.50 D) were included. Following random selection of one eye for analysis (where both eyes were available), a total of 10,488 normal eyes of 10,488 healthy participants were finally included.

### Instrumentation

In the full UK Biobank cohort, VA was measured using a LogMAR chart (precision-vision.com) on a digital screen at 4 m—or 1 m if a participant was unable to read at 4 m (eyes of such participants would have been excluded due to poor VA per the selection process detailed above)—with habitual distance correction in place. The test would terminate when two or more letters were read incorrectly. Refractive error and CR were measured automatically without cycloplegia using a Tomey RC-5000 autorefractor/keratometer (tomey.com). SER was defined as spherical power + 0.5 × cylindrical power, while CR was given by the mean radius of curvature of the steepest and flattest corneal meridians (3 mm from the corneal apex). The mean values of the first five readings of SER and CR were used for analysis. CH, CRF and IOP were measured once for each eye using ORA per a predetermined protocol (biobank.ctsu.ox.ac.uk/crystal/ukb/docs/Intraocularpressure.pdf), with the measurement reattempted if the participant blinked or if the IOP was greater than 24 mmHg. VA was measured first, followed by autorefraction/keratometry and ORA measurements. The right eye was always measured before the left eye.

### Statistical analyses

Multiple linear regression was first fitted using ordinary least squares (OLS) to test the linear association between CH or CRF (separately) and SER (dependent variable), controlling for age, sex, CR and IOP. As discussed earlier, existing research using general linear models (including ANOVA) made the implicit assumption that CH or CRF had a non-varying (linear) relationship with SER across the whole spectrum of myopia. We visually inspected the normal Q−Q and residuals versus fitted plots to assess if this assumption was valid.^38^ A normal Q−Q plot was a graph that ranked the *observed* residuals (i.e., difference between actual and predicted SER) from smallest to largest on the *y*-axis while showing the *theoretical* residuals of the normal distribution (standardised to have zero mean and unit standard deviation, SD) on the *x*-axis. As such, the plot should follow a straight line if the assumption of the normality of residuals was met. The residuals versus fitted plot was a graph that showed the observed residuals on the *y*-axis and the predicted SER on the *x*-axis. It enabled the assumption of the homogeneity of variance (equal variance of residuals across predicted values) to be checked. Violation of these assumptions would suggest that the relationship between CH/CRF and SER could not be modelled linearly across the full range of myopia.^38^ Multicollinearity (strong correlation between two or more explanatory variables) was checked with the variance inflation factor, treating 10 as the cut-off.^39^

In contrast to OLS regression, quantile regression (QR) is a much more flexible statistical modelling technique because it allows for variability in the magnitude/direction of association between an independent variable and the dependent variable by modelling the relationship in different conditional quantiles of the dependent variable.^40^ In other words, it does not assume the relationship to be linear across the entire phenotypic distribution of the dependent variable. Note that a quantile is just the decimal form of a percentile. For example, 0.5 quantile is the decimal representation of the 50th percentile or median. ‘Conditional’ means that the quantisation of the dependent variable is conditional on the explanatory variables. QR was previously used to show that various genetic and environmental risk factors for myopia had larger effects in children with higher myopia.^41^ In this work, we applied QR to assess if the magnitude of association between CH/CRF and SER (controlling for the same covariates as OLS regression) differed across 49 equally spaced conditional quantiles of SER using the *Quantreg* package in R version 4.2.2 (R Core Team 2022, r-project.org), ranging from 0.002 (most myopic quantile) to 0.998 (almost emmetropic). CH, CRF and continuous covariates were standardised (zero mean and unit SD) in OLS regression and QR. The significance level was set to 0.05 and the R code is openly available at https://github.com/fyii200/cornealBiomechanics.

## RESULTS

Of the 10,488 included participants with a mean (SD; range) age of 53 (8; 40–69) years, 5819 were female and 4669 were male. The majority of these participants were self-reportedly ‘British’ (*N* = 8720), ‘Irish’ (*N* = 320) or of ‘any other white background’ (*N* = 559). The mean (SD; range) SER, VA, CH, CRF, IOP and CR were −2.91 (2.20; −0.50 to −22.99) D, −0.10 (0.07; −0.48 to 0.00) LogMAR, 10.7 (1.8; 4.8–17.5) mmHg, 10.8 (1.9; 5.4–17.9) mmHg, 15.8 (3.4; 7.5–28.4) mmHg and 7.7 (0.3; 7.1–8.6) mm, respectively.

**FIGURE 2:**
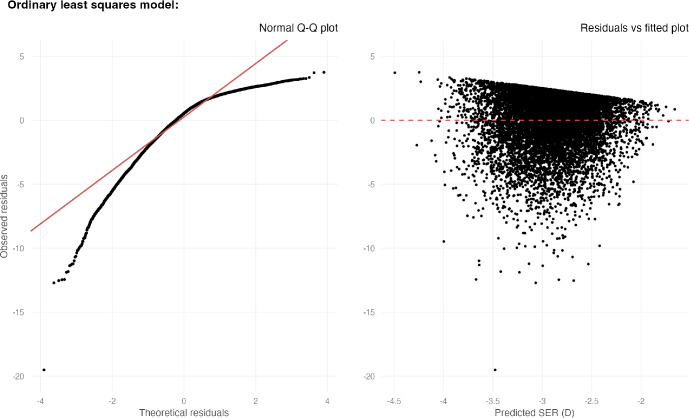
Normal Q−Q plot (left) and residuals versus fitted plot (right) derived from the corneal hysteresis (CH) multiple linear regression model. The residuals can be assumed to be normally distributed if the normal Q−Q plot follows the diagonal straight line (red). The variance of residuals can be assumed to be homogeneous/equal across the fitted values if the points are evenly spread out along the *y*-axis in the residuals versus fitted plot. These diagnostic plots strongly suggest that the linearity assumption is violated. Note that CH had a very strong linear correlation with corneal resistance factor (CRF; Pearson correlation coefficient: 0.85), so the diagnostic plots for CRF are the same as that shown in the figure above. SER, spherical equivalent refraction.

OLS multiple linear regression suggested that every SD decrease in CH and CRF was associated with 0.08 D (95% CI: 0.04–0.12; *p* < 0.001) and 0.10 D (0.04–0.15 D; *p* < 0.001) of higher myopia, respectively (Table 1). IOP was observed to increase with greater myopia. There was no issue of multicollinearity in both regression models (variance inflation factors were smaller than two for all variables). However, residual analysis strongly indicated that the relationship between CH/CRF and SER could not be well modelled using OLS regression across the full range of myopia: the residuals were not normally distributed (left plot in Figure 2) and had unequal variance across the range of predicted SER (right plot in Figure 2).

**TABLE 1:** Ordinary least squares multiple linear regression describing the association between corneal hysteresis (CH; left) or corneal resistance factor (CRF; right) and spherical equivalent refraction (dependent variable), controlling for age, sex (female as reference), intraocular pressure (IOP) and corneal radius of curvature (CR).

Explanatory	Estimates	95% CI	*p*	Explanatory	Estimates	95% CI	*p*
Intercept	−2.95	−3.00 to −2.89	<0.001	Intercept	−2.95	−3.00 to −2.89	<0.001
CH	0.08	0.04 to 0.12	<0.001	CRF	0.10	0.04 to 0.15	<0.001
CR	0.23	0.18 to 0.27	<0.001	CR	0.23	0.18 to 0.27	<0.001
IOP	−0.26	−0.30 to −0.22	<0.001	IOP	−0.31	−0.36 to −0.26	<0.001
Age	0.16	0.12 to 0.20	<0.001	Age	0.16	0.12 to 0.20	<0.001
Male	0.08	−0.01 to 0.16	0.08	Male	0.08	−0.01 to 0.16	0.08

*Note*: Estimates (beta coefficients) are standardised to have zero mean and unit standard deviation.

**FIGURE 3:**
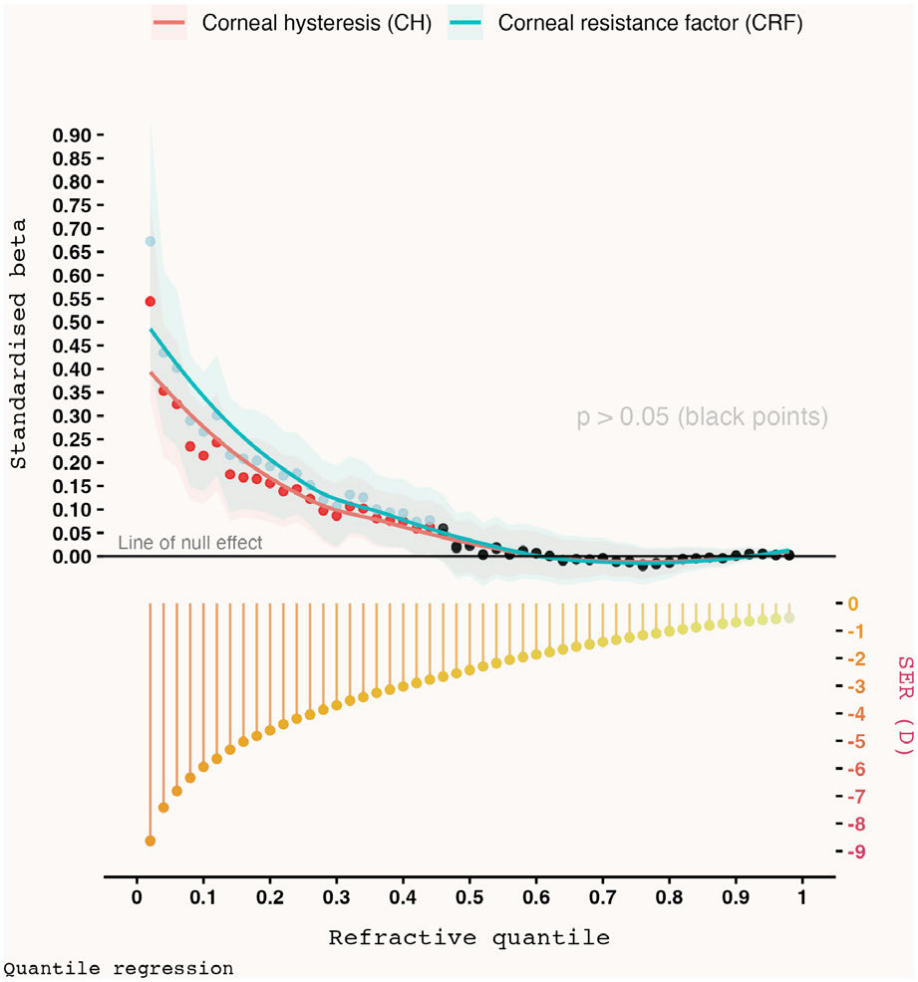
Standardised beta coefficients describing the magnitude of association between corneal hysteresis or corneal resistance factor and spherical equivalent refraction (SER), controlling for age, sex, intraocular pressure and corneal radius of curvature, across different conditional quantiles (top) of SER (bottom). Shaded area represents 95% CI.

In line with this, QR pointed towards a varying, not constant, magnitude of association between CH/CRF and SER across the full range of myopia (Figure 3 and Table 2). There was little statistical evidence that either corneal biomechanical property was associated with SER from around −0.53 to −2.67 D. However, a positive association became evident from the quantile corresponding to −2.78 D, though every SD decrease in CH and CRF was only associated with 0.06 D (95% CI: 0.01–0.12; *p* = 0.03) and 0.08 D (95% CI: 0.01–0.15; *p* = 0.03) higher myopia, respectively, which was not too different from that derived from OLS (linear) regression. Of note, the estimates derived from QR were *drastically different* from OLS regression thereafter, as the magnitude of association was found to increase exponentially with increasing myopia (Figure 3). To illustrate, in the quantile corresponding to around −5.00 D, the magnitude increased to 0.17 D (95% CI: 0.08–0.25; *p* < 0.001) and 0.21 D (95% CI: 0.10–0.31; *p* < 0.001) higher myopia for every SD decrease in CH and CRF. In the most myopic quantile (around −8.50 D), this further increased to 0.54 D (95% CI: 0.33–0.76; *p* < 0.001) and 0.67 D (95% CI: 0.41–0.93; *p* < 0.001) higher myopia per SD decrease in CH and CRF. We additionally performed a sensitivity analysis by repeating the QR analysis after removing 747 eyes with ocular hypertension (IOP > 21 mmHg), and the results were found to be consistent with the primary analysis (Appendix S1).

**TABLE 2:** Standardised beta coefficients describing the magnitude of association between corneal hysteresis or corneal resistance factor and spherical equivalent refraction (SER), controlling for age, sex, intraocular pressure and corneal radius of curvature, across different conditional quantiles of myopia.

Quantile	SER	Corneal hysteresis			Corneal resistance factor		
		Standardised beta	SE	*p*	Standardised beta	SE	*p*
0.02	−8.63	0.544	0.109	<0.001	0.672	0.134	<0.001
0.04	−7.42	0.353	0.072	<0.001	0.435	0.089	<0.001
0.06	−6.82	0.325	0.068	<0.001	0.402	0.085	<0.001
0.08	−6.34	0.235	0.059	<0.001	0.290	0.072	<0.001
0.10	−5.94	0.215	0.053	<0.001	0.266	0.065	<0.001
0.12	−5.65	0.243	0.053	<0.001	0.301	0.065	<0.001
0.14	−5.32	0.175	0.051	<0.001	0.216	0.063	<0.001
0.16	−5.03	0.168	0.044	<0.001	0.208	0.054	<0.001
0.18	−4.82	0.165	0.044	<0.001	0.204	0.054	<0.001
0.20	−4.62	0.156	0.042	<0.001	0.193	0.052	<0.001
0.22	−4.40	0.139	0.039	<0.001	0.172	0.048	<0.001
0.24	−4.20	0.143	0.036	<0.001	0.177	0.044	<0.001
0.26	−4.05	0.123	0.035	<0.001	0.152	0.043	<0.001
0.28	−3.87	0.098	0.035	0.005	0.120	0.043	0.01
0.30	−3.71	0.086	0.036	0.01	0.107	0.044	0.02
0.32	−3.54	0.107	0.034	0.002	0.132	0.042	0.002
0.34	−3.41	0.102	0.032	0.002	0.126	0.040	0.002
0.36	−3.26	0.081	0.031	0.009	0.100	0.038	0.009
0.38	−3.14	0.076	0.030	0.01	0.094	0.037	0.01
0.40	−3.03	0.074	0.030	0.01	0.092	0.037	0.01
0.42	−2.90	0.060	0.030	0.05	0.074	0.037	0.05
0.44	−2.78	0.062	0.029	0.03	0.077	0.036	0.03
0.46	−2.67	0.048	0.030	0.10	0.059	0.037	0.10
0.48	−2.55	0.017	0.029	0.54	0.022	0.035	0.54
0.50	−2.43	0.022	0.029	0.45	0.027	0.035	0.45
0.52	−2.30	0.003	0.028	0.91	0.004	0.034	0.91
0.54	−2.18	0.016	0.027	0.56	0.019	0.033	0.57
0.56	−2.06	0.004	0.025	0.88	0.005	0.031	0.88
0.58	−1.96	0.010	0.024	0.69	0.012	0.030	0.69
0.60	−1.87	0.006	0.023	0.79	0.007	0.029	0.81
0.62	−1.78	0.001	0.023	0.98	0.001	0.028	0.98
0.64	−1.68	−0.008	0.022	0.72	−0.010	0.027	0.72
0.66	−1.58	−0.006	0.021	0.79	−0.007	0.026	0.79
0.68	−1.50	−0.007	0.020	0.74	−0.008	0.024	0.74
0.70	−1.41	−0.004	0.018	0.84	−0.004	0.022	0.84
0.72	−1.33	−0.010	0.018	0.58	−0.012	0.022	0.58
0.74	−1.25	−0.010	0.017	0.54	−0.013	0.021	0.54
0.76	−1.17	−0.017	0.017	0.31	−0.021	0.020	0.31
0.78	−1.10	−0.014	0.015	0.35	−0.017	0.018	0.34
0.80	−1.02	−0.011	0.013	0.39	−0.014	0.017	0.40
0.82	−0.95	−0.005	0.012	0.65	−0.007	0.014	0.65
0.84	−0.88	−0.004	0.011	0.71	−0.005	0.013	0.71
0.86	−0.81	−0.002	0.009	0.80	−0.003	0.012	0.80
0.88	−0.75	−0.004	0.008	0.65	−0.005	0.010	0.65
0.90	−0.70	0.001	0.007	0.86	0.002	0.009	0.86
0.92	−0.66	0.004	0.006	0.49	0.005	0.008	0.49
0.94	−0.61	0.005	0.005	0.36	0.006	0.006	0.36
0.96	−0.57	0.002	0.004	0.50	0.003	0.005	0.50
0.98	−0.53	0.002	0.002	0.31	0.003	0.003	0.31

Abbreviation: SE, standard error.

## DISCUSSION

Using QR, we found evidence that CH and CRF varied in a highly non-linear fashion with SER. The biomechanics of the cornea appeared compromised (reduced ability to dissipate energy and resist deformation) from around −3.00 D, a level much lower than previously thought. These biomechanical changes were observed to become exponentially—not linearly as assumed by earlier studies—more pronounced with increasing myopia. Importantly, we showed that by erroneously assuming a linear relationship between CH/CRF and SER across the full range of myopia severity, OLS regression progressively (increasingly so with more negative SER) underestimated the true extent of corneal biomechanical weakening in myopia, as it failed to capture the intricate variations in CH and CRF across different quantiles of SER.

The findings that corneal biomechanics appear compromised from around −3.00 D and, exponentially so thereafter, bear a remarkable resemblance to the trend lines describing the prevalence of pathologic myopia across different levels of refractive error in the population-based Handan Eye Study (Chinese)^33^ and Blue Mountains Eye Study (mostly White Australians).^34^ In these studies, while the prevalence of pathologic myopia was close to 0% in myopia less than −3.00 D, it increased to 3%–5% around −4.00 D, and then to 11% around −5.00 D, before reaching over 50% around −8.00 D. Such a trend also appears consistent with the non-linear increase in the odds of retinal detachment with more negative SER.^35^ As with most viscoelastic collagenous tissues, the cornea exhibits a non-linear creep response in that its rate of deformation increases with greater levels of applied stress.^42^ This may explain why the cornea appears to weaken exponentially with increasing myopia, as the shear stress stemming from posterior chamber expansion during myopic eye growth may also act on the anterior segment epiphenomenally. Indeed, animal models of myopia demonstrated that non-uniform vitreous chamber expansion induced by hemi-retinal form deprivation (i.e., diffuser applied to half of the visual field) or anisotropic optical defocus (i.e., use of a cylindrical lens to impose varying degrees of defocus in different meridians) could give rise to similar corneal changes anteriorly in terms of the axis of astigmatism.^1^ Collectively, these findings support the notion that corneal biomechanics (measured by ORA) may be indictive of the overall biomechanics of the globe, though more in vivo work is still required to corroborate this.

Although previous studies (cited in the introduction) generally found evidence of reduced CH and/or CRF in high myopia, a smaller number of studies failed to find a similar association for both CH and CRF.^16,25,26,28^ While differences in age (children vs. adults), statistical models (adjusted covariates) and sample sources (derived from the general population or a less general, clinical setting) may account for some of the discrepancies in results, the present study suggests that statistical power might have also played an important (if not dominant) role. Considering that there is virtually no association between CH/CRF and SER up to around −3.00 D, studies assuming linearity across the full range of myopia would either need to have a large overall sample size or a sufficient number of eyes with extreme myopia, or both, to reject the null hypothesis. Indeed, studies that failed to find a statistically significant association in adults (or in a predominantly adult sample) usually had a relatively small sample size ranging from 43 to 117 participants and a narrower range of myopia (myopia up to −9.00 or −11.00 D)^25,26,28^ compared with studies^15,18–23,29^ that reported a significant result, which generally had a larger sample size (64–651 participants; *N* ≥ 135 in four studies) and included more extreme myopia, with a minimum SER ranging from −12.00 to −29.00 (mean ± 2 SD when the range was not reported).

The present work is the first in-depth analysis of corneal biomechanical changes across different levels of myopia using QR, a flexible statistical modelling technique different from the general linear models employed by previous studies.^14,15,18–24,27,29^ This, coupled with the use of a very large sample (largest among all pertinent studies) derived from a richly phenotyped, population-based cohort of healthy adults with a wide range of myopia (−0.50 to −22.99 D), enabled us to shed light on the nature of normative (i.e., without the influence of ocular and systemic comorbidities) CH and CRF changes across the full range of myopia. The main limitation of the work is the lack of axial length data in the UK Biobank. A similar analysis using axial length would have been even more meaningful, considering that it is more reflective of the extent of ocular stretching, not to mention there is some evidence that its relationship with refractive error is not perfectly linear.^43,44^ Although refractive error was determined objectively under non-cycloplegic conditions, this is unlikely to have any practically important influence on our overall findings because the amplitude of accommodation can be assumed to be minimal in our sample of older adults aged 40 years and above.

In conclusion, corneal biomechanical weakening is evident in myopia from around −3.00 D. This was observed to become exponentially, not linearly, more pronounced with increasing myopia. These findings collectively indicate that structural sequelae of myopia are not specific to high myopia and underscore the importance of not misconstruing the dioptric threshold of −6.00, or −5.00 D for that matter, as being intrinsically dichotomous (safe vs. unsafe threshold).

## Supplementary Information

S1: Quantile regression was repeated after removing 747 eyes with ocular hypertension (intraocular pressure > 21mmHg but not diagnosed with glaucoma), leaving 9741 eyes for analysis.

## Data Availability

This research was conducted using data from the UK Biobank under project ID 90655. Data directly supporting the results of this work are only available to the immediate research team members due to UK Biobank's access control policy. Bona fide researchers can, however, apply for access at https://www.ukbiobank.ac.uk/enable-your-research/apply-for-access. The R script used to perform the analyses described in this work are freely available at https://github.com/fyii200/cornealBiomechanics.
