# Supplementary material for: Corneal biomechanics are not exclusively compromised in high myopia
Source: Ophthalmic Physiol Opt. 2024 Apr 2;44(5):977–86. doi: 10.1111/opo.13313 (PMC12862013; doi:10.1111/opo.13313)
Supplement: Supplementary file 1 — S1: Quantile regression was repeated after removing 747 eyes with ocular hypertension (intraocular pressure > 21mmHg but not diagnosed with glaucoma), leaving 9741 eyes for analysis. [file 44402_2024_4405018_MOESM1_ESM.docx]

**S1: Quantile regression was repeated after removing 747 eyes with ocular hypertension (intraocular pressure > 21mmHg but not diagnosed with glaucoma), leaving 9741 eyes for analysis.**

The coefficient plot below shows the standardised beta coefficients describing the magnitude of association between corneal hysteresis or corneal resistance factor and spherical equivalent refraction (SER), controlling for age, sex, intraocular pressure and corneal radius of curvature, across different conditional quantiles (top) of SER (bottom). Shaded area represents 95% CI.

Standardised beta coefficients across different conditional quantiles of myopia in tabular format. “SER” refers to spherical equivalent refraction, while “SE” refers to standard error.

| Quantile | SER | Corneal hysteresis | | | Corneal resistance factor | | |
| --- | --- | --- | --- | --- | --- | --- | --- |
|  |  | ***Standardised beta*** | ***SE*** | ***P*** | ***Standardised beta*** | ***SE*** | ***P*** |
| 0.02 | -8.56 | 0.548 | 0.109 | <0.001 | 0.645 | 0.129 | <0.001 |
| 0.04 | -7.35 | 0.335 | 0.071 | <0.001 | 0.397 | 0.084 | <0.001 |
| 0.06 | -6.78 | 0.327 | 0.073 | <0.001 | 0.387 | 0.086 | <0.001 |
| 0.08 | -6.25 | 0.214 | 0.061 | <0.001 | 0.255 | 0.073 | <0.001 |
| 0.10 | -5.91 | 0.210 | 0.052 | <0.001 | 0.248 | 0.061 | <0.001 |
| 0.12 | -5.61 | 0.221 | 0.055 | <0.001 | 0.262 | 0.066 | <0.001 |
| 0.14 | -5.28 | 0.167 | 0.053 | 0.002 | 0.198 | 0.063 | 0.002 |
| 0.16 | -5.01 | 0.152 | 0.045 | <0.001 | 0.180 | 0.054 | <0.001 |
| 0.18 | -4.79 | 0.170 | 0.045 | <0.001 | 0.201 | 0.053 | <0.001 |
| 0.20 | -4.57 | 0.173 | 0.043 | <0.001 | 0.203 | 0.051 | <0.001 |
| 0.22 | -4.36 | 0.140 | 0.040 | <0.001 | 0.166 | 0.047 | <0.001 |
| 0.24 | -4.18 | 0.152 | 0.036 | <0.001 | 0.180 | 0.042 | <0.001 |
| 0.26 | -4.04 | 0.145 | 0.036 | <0.001 | 0.171 | 0.043 | <0.001 |
| 0.28 | -3.84 | 0.103 | 0.036 | 0.004 | 0.122 | 0.042 | 0.004 |
| 0.30 | -3.67 | 0.087 | 0.037 | 0.018 | 0.103 | 0.044 | 0.018 |
| 0.32 | -3.51 | 0.107 | 0.035 | 0.002 | 0.127 | 0.041 | 0.002 |
| 0.34 | -3.38 | 0.110 | 0.034 | 0.001 | 0.130 | 0.040 | 0.001 |
| 0.36 | -3.24 | 0.096 | 0.032 | 0.003 | 0.114 | 0.038 | 0.003 |
| 0.38 | -3.12 | 0.084 | 0.032 | 0.009 | 0.100 | 0.038 | 0.008 |
| 0.40 | -3.01 | 0.083 | 0.031 | 0.008 | 0.098 | 0.037 | 0.008 |
| 0.42 | -2.88 | 0.078 | 0.030 | 0.010 | 0.092 | 0.036 | 0.011 |
| 0.44 | -2.75 | 0.072 | 0.030 | 0.017 | 0.086 | 0.036 | 0.017 |
| 0.46 | -2.64 | 0.048 | 0.030 | 0.106 | 0.057 | 0.035 | 0.106 |
| 0.48 | -2.52 | 0.035 | 0.030 | 0.241 | 0.042 | 0.035 | 0.241 |
| 0.50 | -2.39 | 0.024 | 0.030 | 0.417 | 0.029 | 0.036 | 0.416 |
| 0.52 | -2.26 | 0.022 | 0.030 | 0.457 | 0.026 | 0.035 | 0.457 |
| 0.54 | -2.14 | 0.026 | 0.028 | 0.359 | 0.030 | 0.033 | 0.359 |
| 0.56 | -2.02 | 0.018 | 0.026 | 0.479 | 0.022 | 0.031 | 0.480 |
| 0.58 | -1.92 | 0.023 | 0.025 | 0.352 | 0.027 | 0.029 | 0.352 |
| 0.60 | -1.83 | 0.017 | 0.023 | 0.458 | 0.021 | 0.028 | 0.459 |
| 0.62 | -1.75 | 0.017 | 0.023 | 0.456 | 0.021 | 0.028 | 0.457 |
| 0.64 | -1.66 | 0.009 | 0.022 | 0.684 | 0.011 | 0.026 | 0.683 |
| 0.66 | -1.56 | 0.010 | 0.021 | 0.639 | 0.012 | 0.025 | 0.640 |
| 0.68 | -1.48 | 0.013 | 0.020 | 0.502 | 0.016 | 0.023 | 0.503 |
| 0.70 | -1.39 | 0.009 | 0.018 | 0.628 | 0.010 | 0.022 | 0.628 |
| 0.72 | -1.32 | 0.009 | 0.018 | 0.624 | 0.010 | 0.021 | 0.623 |
| 0.74 | -1.24 | 0.003 | 0.018 | 0.876 | 0.003 | 0.021 | 0.875 |
| 0.76 | -1.15 | -0.011 | 0.017 | 0.518 | -0.013 | 0.021 | 0.514 |
| 0.78 | -1.07 | 0.003 | 0.015 | 0.829 | 0.004 | 0.018 | 0.829 |
| 0.80 | -1 | -0.002 | 0.014 | 0.872 | -0.003 | 0.016 | 0.872 |
| 0.82 | -0.93 | 0.001 | 0.012 | 0.913 | 0.002 | 0.014 | 0.913 |
| 0.84 | -0.87 | 0.008 | 0.010 | 0.451 | 0.009 | 0.012 | 0.451 |
| 0.86 | -0.81 | 0.009 | 0.010 | 0.348 | 0.011 | 0.011 | 0.351 |
| 0.88 | -0.74 | 0.005 | 0.008 | 0.579 | 0.006 | 0.010 | 0.581 |
| 0.90 | -0.7 | 0.005 | 0.007 | 0.441 | 0.006 | 0.008 | 0.443 |
| 0.92 | -0.65 | 0.008 | 0.006 | 0.187 | 0.010 | 0.007 | 0.188 |
| 0.94 | -0.6 | 0.006 | 0.005 | 0.249 | 0.007 | 0.006 | 0.251 |
| 0.96 | -0.57 | 0.005 | 0.004 | 0.258 | 0.006 | 0.005 | 0.187 |
| 0.98 | -0.53 | 0.002 | 0.002 | 0.330 | 0.003 | 0.003 | 0.330 |
